# Temperature but not ocean acidification affects energy metabolism and enzyme activities in the blue mussel, *Mytilus edulis*

**DOI:** 10.1002/ece3.7289

**Published:** 2021-03-05

**Authors:** Omera B. Matoo, Gisela Lannig, Christian Bock, Inna M. Sokolova

**Affiliations:** ^1^ School of Biological Sciences University of Nebraska‐Lincoln Lincoln NE USA; ^2^ Department of Biological Sciences University of North Carolina at Charlotte Charlotte NC USA; ^3^ Helmholtz Centre for Polar and Marine Research Alfred Wegener Institute Bremerhaven Germany; ^4^ Department of Marine Biology Institute of Biological Sciences Universität Rostock Rostock Germany

**Keywords:** energy metabolism, global warming, *Mytilus*, NMR‐based metabolomics, ocean acidification

## Abstract

1. In mosaic marine habitats, such as intertidal zones, ocean acidification (OA) is exacerbated by high variability of pH, temperature, and biological CO_2_ production. The nonlinear interactions among these drivers can be context‐specific and their effect on organisms in these habitats remains largely unknown, warranting further investigation.
2. We were particularly interested in *Mytilus edulis* (the blue mussel) from intertidal zones of the Gulf of Maine (GOM), USA, for this study. GOM is a hot spot of global climate change (average sea surface temperature (SST) increasing by >0.2°C/year) with >60% decline in mussel population over the past 40 years.
3. Here, we utilize bioenergetic underpinnings to identify limits of stress tolerance in *M. edulis* from GOM exposed to warming and OA. We have measured whole‐organism oxygen consumption rates and metabolic biomarkers in mussels exposed to control and elevated temperatures (10 vs. 15°C, respectively) and current and moderately elevated *P*_CO2_ levels (~400 vs. 800 µatm, respectively).
4. Our study demonstrates that adult *M. edulis* from GOM are metabolically resilient to the moderate OA scenario but responsive to warming as seen in changes in metabolic rate, energy reserves (total lipids), metabolite profiles (glucose and osmolyte dimethyl amine), and enzyme activities (carbonic anhydrase and calcium ATPase).
5. Our results are in agreement with recent literature that OA scenarios for the next 100–300 years do not affect this species, possibly as a consequence of maintaining its in vivo acid‐base balance.

## Graphical abstract

Our study demonstrates that adult *Mytilus edulis* from Gulf of Maine, one of the hot spots for global climate change, are metabolically resilient to the moderate acidification scenarios for the next 100–300 years but responsive to warming as seen in changes in metabolic rate, energy reserves, metabolite profiles, and enzyme activities.

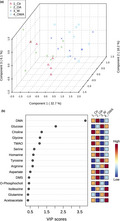

## 1 INTRODUCTION

Continued increase in atmospheric CO_2_ and its subsequent uptake by oceans profoundly affects marine ecosystems (IPCC, 2014). Changes experienced by organisms include an increase in the global sea surface temperature (SST) and oceans’ partial pressure of CO_2_ (*P*_CO2_), which leads to ocean acidification (OA) (Doney et al., 2012). Climate change models predict an average increase of 1.8–4.0°C (with some estimates as high as 6.4°C) and a decline by 0.3–0.4 pH units by the year 2100, depending on the CO_2_ emission scenario (IPCC, 2014). Warming and OA can negatively impact marine organisms (Kroeker et al., 2013, 2014). However, in mosaic habitats such as intertidal and coastal zones, the outcome of multiple drivers, including warming and OA, is complicated by context‐specific and nonlinear interactions among the drivers (Gunderson et al., 2016 and references therein) so that the net effect could be additive, antagonistic, or synergistic (Todgham & Stillman, 2013). The species’ response to interactive effects of warming and OA in such environments remains largely unknown and warrants further investigation (Gunderson et al., 2016).

Temperature is a key variable that affects physiology, survival, and distribution of ectotherms (Kroeker et al., 2013, 2014). Deviation of temperature from the optimum results in disturbance of energy balance and decrease in aerobic scope of organisms (Pörtner, 2012; Sokolova, 2013; Sokolova et al., 2012). OA negatively affects survival, metabolism, calcification, growth, reproduction, and immune responses across a range of marine taxa (Kroeker et al., 2013). Elevated *P*_CO2_ shifts the acid–base balance of organisms (Melzner et al., 2009) and most calcifiers have limited capacity to counteract OA‐induced extracellular acidosis (Pörtner, 2008). This in turn can increase the energy costs to maintain cellular and organismal homeostasis in animals (Ivanina et al., 2020; Pörtner, 2008; Sokolova et al., 2012; Stapp et al., 2018; Stumpp et al., 2012).

Responses of marine molluscs to OA are highly variable (Sokolova et al., 2015 and references therein). Inter‐ and intrapopulation variability in OA sensitivity has been shown depending on habitat, scales of environmental variability, and other concomitant stressors (Parker et al., 2011; Stapp et al., 2017; Vargas et al., 2017; Waldbusser et al., 2015). In mosaic environments, local adaptation, as well as temporally and spatially varying selection, can select for metabolically plastic, stress‐tolerant genotypes that can maintain optimal phenotypes (including energetic sustainability) in a broad range of environmental conditions. Importantly, bioenergetic responses can predict tolerance limits under environmentally realistic scenarios of stress exposure (Sokolova et al., 2012) providing a common denominator to integrate responses to multiple stressors. Quantifying the independent and interactive effects of multiple stressors to identify metabolic tipping points is essential to determine the impact of global climate change on marine organisms and ecosystems (Boyd & Brown, 2015).

This study aims to determine the interactive effects of elevated temperature and *P*_CO2_ on energy metabolism and biomineralization‐related enzymes in an ecologically and economically important bivalve mollusk, the blue mussel *Mytilus edulis* Linnaeus 1758. It is a critical foundation species in coastal ecosystems (Seed, 1969) that increasingly faces risk of local extinction along the USA east coast *(*Jones et al., 2009; Sorte et al., 2017). We were particularly interested in mussel populations from intertidal zones of Gulf of Maine (GOM), USA, for this study. GOM is a hot spot of global climate change with >60% (range 29%–100%) decline in mussel population over the past 40 years (Sorte et al., 2017). Within GOM, the previous decade has witnessed an average warming of >0.2°C/year (Sorte et al., 2017). Satellite observations of SST within the GOM show that the region was warming at a faster rate than 99% of the global ocean (Pershing et al. 2015), with the highest average annual values exceeding over 150 years of observations held in NOAA’s Merged Land‐Ocean Surface Temperature Analysis database (Salisbury & Jönsson, 2018). Combined with poor buffering capacity of the seawater in the coastal GOM regions (Ekstrom et al., 2015), temperature alone produces an annual change of 0.013 in pH and 1.06 in Ω_AR_ (Salisbury & Jönsson, 2018). Modeling data indicate that these rapid changes in the GOM together with physical processes in the area (e.g., strong tides, wind‐driven mixing, coastal currents, and adjacent equilibrium with Northwestern Atlantic) alter ocean carbonate parameters but also mitigate the decrease, or even raise pH (Salisbury & Jönsson, 2018). Mussels inhabiting GOM are thus exposed to one of the fastest rates of warming in the world, in addition to ocean acidification.

The metabolic plasticity of *M. edulis* to the combined effects of elevated *P*_CO2_ and temperature from GOM is not yet fully understood. Here, we utilize the bioenergetic underpinnings of stress physiology to identify limits and mechanisms of stress tolerance in *M. edulis*. We tested the hypothesis that elevated temperature will exacerbate the effects of ocean acidification, resulting in elevated basal energy metabolism, which may decrease the amount of energy reserves and reduce enzyme activities involved in energy‐demanding process of shell formation. To this end, we measured whole‐organism oxygen consumption rates and metabolic biomarkers in mussels exposed to control and moderately elevated temperatures (10 vs. 15°C) and *P*_CO2_ (~400 vs. 800 µatm) to mimic a realistic scenario of ocean warming and acidification. Standard metabolic rate (SMR) represents the basal energy cost for maintenance and is widely used to assess stress response (Pettersen et al., 2018). To account for possible tissue‐specific variation in responses, we conducted a comprehensive analyses of the bioenergetic health index by measuring energy‐related biomarkers (cellular and tissue energy reserves), metabolic profiles, and specific enzyme activities (acid–base regulating enzyme and energy‐demanding ion transport enzymes) in different tissues depending on their physiological role. In mollusks, including *M. edulis*, carbonic anhydrase (CA) is the major acid–base regulating enzyme (Li et al., 2016; Ramesh et al., 2020; Wang et al., 2017). It plays a key role in gas exchange, acid–base regulation, calcification, and ion transport. Given the central role of CA, its activity is likely to play an important part in physiological mechanisms of acclimatization and tolerance of marine calcifiers, including *M. edulis*, to CO_2_‐induced shifts in ocean chemistry as well as to other stressors such as temperature, salinity, or pollution. However, the fundamental information about the tissue distribution and thermal sensitivity of CA kinetics in *M. edulis* is not known. We, therefore, analyzed the tissue distribution and temperature sensitivity (determined by apparent activation energy and Arrhenius breakpoint temperature) of CA activity here in a separate set of experiment. This will provide the much‐needed data about the nuanced role of CA in regulation of acid–base balance and biomineralization in *M. edulis*.

## 2 MATERIALS AND METHODS

### 2.1 Chemicals

Unless otherwise indicated, all chemicals and enzymes were purchased from Sigma‐Aldrich, Roche, or Fisher Scientific and were of analytical grade or higher.

### 2.2 Animal collection, maintenance and experimental design

2.2.1

Blue mussels *M. edulis* were collected from Biddeford Pool, Gulf of Maine (43°26′50.6N, 70°21′19.0W) in early summer 2011 and shipped on ice by an overnight delivery to the University of North Carolina at Charlotte. Mussels were kept in tanks with recirculating artificial seawater (ASW) (Instant Ocean^®^, Kent Marine) at 9.6 ± 0.3°C and 30 ± 1 salinity (practical salinity units, PSU), aerated with ambient air for 10 days. Mussels were then randomly assigned to four treatment groups, and each group was exposed for 4 weeks to one of the four possible combinations of two levels of *P*_CO2_ and two temperatures. The two selected *P*_CO2_ levels were representative of the present‐day conditions (~400 µatm *P*_CO2_; normocapnia) and atmospheric *P*_CO2_ concentrations predicted by a moderate scenario of the Intergovernmental Panel for Climate Change (IPCC) for the year 2100 (~800 µatm *P*_CO2_; hypercapnia) (IPCC, 2014). The temperatures were chosen to represent the average water temperature of mussels at the time of collection (10°C), and a +5°C increase predicted for the year 2100 by an IPCC scenario 'business‐as‐usual' (15°C). Both experimental temperatures are within the environmentally relevant range for the studied mussel population in the GOM. For the bivalves exposed to elevated temperature, water temperature in the tanks was slowly raised from 10°C by 1°C per day until 15°C was achieved and the experimental exposures began.

Two replicate tanks were set for each experimental treatment. Water in normocapnic treatments was bubbled with ambient air whereas for hypercapnic treatments ambient air was mixed with 100% CO_2_ (Roberts Oxygen, Charlotte, NC,USA) using precision mass flow controllers (Cole‐Parmer, Vernon Hills, IL, USA). The air‐CO_2_ mixture flow rate was set up to maintain the respective systems at a steady‐state pH. Animals were fed *ad libitum* on alternative days with 2 ml per tank of commercial algal mixture containing *Isochrysis* spp., *Pavlova* spp., *Thalassoisira weissflogii*, and *Tetraselmis* spp. with 5–20 μm cells (Shellfish Diet 1800, Reed Mariculture, Campbell, CA, USA). Mortality was checked daily and animals that gaped and did not respond to a mechanical stimulus were recorded as dead and immediately removed. Artificial Sea Water (ASW) for all exposures were prepared using the same batch of Instant Ocean^®^ (Kent Marine, Acworth, GA, USA) salt to avoid potential variations in water chemistry. Carbonate chemistry of seawater was determined periodically during experimental exposures as described elsewhere (Beniash et al., 2010). Seawater temperature and chemistry data are shown in Table 1.

**TABLE 1:** Summary of water chemistry parameters during experimental exposure

Parameters	Assay temperature			
	10°C		15°C	
	Normocapnia	Hypercapnia	Normocapnia	Hypercapnia
Salinity, PSU	30.04 ± 0.07	30.50 ± 0.03	30.15 ± 0.04	30.14 ± 0.05
Temperature, °C	9.51 ± 0.06	9.76 ± 0.02	14.95 ± 0.01	14.99 ± 0.01
pH_NBS_	8.13 ± 0.00	7.91 ± 0.00	8.18 ± 0.00	7.92 ± 0.00
*P*_CO2_, µatm	585.39 ± 39	1,026.97 ± 15.07	536.14 ± 4.35	1,069.41 ± 13.90
HCO_3_^−^, (μmol/kg SW)	2,735.82 ± 2.91	2,863.82 ± 3.49	2,628.49 ± 2.73	2,823.22 ± 2.89
CO_3_^2−^ (μmol/kg SW)	155.50 ± 1.23	100.37 ± 1.49	202.67 ± 1.18	118.56 ± 1.24
CO_2_ (μmol/kg SW)	26.85 ± 0.26	46.42 ± 0.67	20.66 ± 0.16	41.03 ± 0.53
Ω_calcite_	3.80 ± 0.02	2.44 ± 0.03	4.97 ± 0.02	2.91 ± 0.03
Ω_aragonite_	2.38 ± 0.01	1.54 ± 0.02	3.15 ± 0.01	1.84 ± 0.01

Note

Salinity, temperature, pH_NBS,_ and dissolved inorganic carbon (DIC) were measured in water samples collected during the exposure. Average DIC was 2,953.75 ± 111.28 μmol/kg SW. Other parameters are calculated using co2SYS. Data are represented as means ± *SEM*. The same batch of seawater was used throughout the course of the experiment with an average total alkalinity (TA) of 3,098.40 mmol/kg SW. *N* = 5 for DIC and *N* = 36–77 for other parameters.

### 2.3 Standard metabolic rate

Standard metabolic rate was measured as resting oxygen consumption ($\dot{M}$O_2_) of mussels at their respective acclimation temperature and *P*_CO2_ using microfiber optic oxygen probes (Tx‐Type, PreSens GmbH, Germany, www.presens.de) as described in Matoo et al., 2013. Two‐point calibration was performed at each temperature and *P*_CO2_ concentration. Mussels were placed into flow‐through respiration chambers and allowed to recover overnight. To avoid interference with postprandial metabolism and feces excretion, animals were fasted for 24 hr prior to the start of $\dot{M}$O_2_ recordings. Water flow (20–25 ml/min) was adjusted so that animals consumed less than 25% of O_2_ at all times to avoid potential inhibitory effects of low oxygen levels on respiration rate. 10 biological replicates for each temperature and *P*_CO2_ group were randomized across chambers and respirometry runs. After each individual run for ~12 hr, the animals were dissected to determine wet tissue mass. Dry tissue mass was calculated from the wet tissue mass assuming an average water content of 80%.

SMR was calculated as follows:

$$\text{SMR}=\frac{\Delta P_{O2}\times \beta_{O2}\times V_{\text{fl}}}{M^{0.8}}$$

SMR—oxygen consumption (μmol O_2_ g^−1^ dry mass h^−1^) normalized to 1 g dry mass, Δ*P*_O2_—difference in partial pressure between in‐ and out‐flowing water (kPa), *β*_O2_—oxygen capacity of water (μmol O_2_ L^−1^ kPa^−1^), Vfl—flow rate (L/h), *M*—dry tissue mass (g) and 0.8—allometric coefficient (Bougrier et al., 1995).

After acclimation, a subset of mussels was dissected, tissues shock‐frozen, and stored in liquid nitrogen for analyses of energy reserves and enzyme activities. Due to limited amount of tissues, we divided samples for different assays depending on the physiological function of a given tissue. The energy reserves (lipids, glycogen, and/or adenylates) were measured in hepatopancreas and adductor muscle that serve as reserve storage sites in bivalves (Cappello et al., 2018). Metabolite profiles were explored by untargeted metabolomics in two metabolically active aerobic tissues, the gills, and the muscle. The effects of warming and OA on biomineralization were assessed by the activities of three key enzymes involved in shell formation (carbonic anhydrase (CA), plasma membrane calcium (Ca^2+^) ATPase, and proton (H^+^) ATPase) in the mantle tissue as the main organ involved in shell formation.

### 2.4 Energy reserves

Lipid content was determined using the chloroform extraction method as described in Ivanina et al., 2013. Concentration of lipids was expressed as g/g wet tissue mass. Concentrations of glycogen and adenylates (μmol g^− 1^ wet tissue mass) were measured using standard NADH‐ or NADPH‐linked spectrophotometric tests described in Ivanina et al., 2013. Adenylate energy charge (AEC) was calculated as follows:

$$\text{AEC}=\frac{\left[\text{ATP}]+0.5\times [\text{ADP}\right]}{\left[\text{ATP}]+[\text{ADP}]+[\text{AMP}\right]}$$

### 2.5 Metabolic profiling based on ^1^H‐NMR spectroscopy

Samples of the muscle and gill tissues were extracted as described elsewhere for untargeted metabolic profiling using NMR spectroscopy (Dickinson et al., 2012; Lannig et al., 2010). Frozen tissues were homogenized under the liquid nitrogen and extracted with ice‐cold perchloric acid (PCA, 0.6 M). Samples were homogenized by ultrasonic treatment (0°C, 360 W) and centrifuged (0°C, 2 min, 16,000 *g*) to remove precipitated protein. Supernatants were neutralized to pH 7.0–7.5 using potassium hydroxide and centrifuged to remove precipitated potassium perchloride. Extracts were freeze‐dried and shipped on dry ice to the Alfred Wegener Institute (Bremerhaven, Germany) for NMR‐based metabolic profiling. Untargeted metabolic profiling using ^1^H‐NMR spectroscopy was performed using a method modified from Schmidt et al. 2017. Samples were dissolved in D_2_O containing 1% trimethylsilylpropanoic acid (TSP) as internal standard to achieve a constant extract/TSP ratio. Typically, a sample volume of 70 µl was transferred to a HRMAS rotor and placed into a triple tuned high‐resolution magic angle spinning (HRMAS) probe of a wide‐bore 400 MHz NMR spectrometer (9.4T Bruker Avance III HD, Bruker Biospin). NMR spectra were collected at a temperature of 4°C and a spinning rate of 3,000 Hz. A set of 1D‐1H‐NMR spectra were collected from every sample including a classical one‐pulse with water saturation protocol (Bruker's zgpr), a Carr–Purcell–Meiboom–Gill (cpmg) sequence used for quantification, a NOESY protocol and a J‐resolved (JRES) protocol for signal identification with parameters as described in (Schmidt et al. 2017). For each sample, 32 scans with a spectral width of 5,000 Hz were collected in 64K data points. The measuring protocol lasted ~28 min per sample. ^1^H‐NMR spectra from the cpmg protocol were used to analyze the metabolic profiles. Spectra were processed with an exponential multiplication (lb = 0.3) and automatically phase‐ and baseline‐corrected using Topsin 3.2 (Bruker Biospin). A total of 24 metabolites was identified from processed tissue spectra and quantified using Chenomx NMR suite 8.1 (Chenomx Inc.). Metabolic profiles were analyzed and tested for statistically significant changes between groups using MetaboAnalyst 4.0 (Chong et al. 2019).

### 2.6 Enzyme activities

Activities of biomineralization‐related enzymes were measured in the mantle edge tissue (a 2–3 mm wide edge along the ventral shell margin functionally specialized for biomineralization) of *M. edulis* exposed to different temperature and *P*_CO2_ conditions. Mantle edge expresses biomineralization‐related genes and is involved in shell deposition in mussels and other bivalves (Bjärnmark et al., 2016; Gazeau et al., 2014). Protein concentrations were determined using Bradford assay (Bradford, 1976) and used to standardize enzyme activities.

#### 2.6.1 Carbonic anhydrase (CA)

For assessment of carbonic anhydrase (carbonate hydrolyase, EC 4.2.1.1) activity, mantle edge tissue was homogenized as described in Ivanina et al., 2013. CA activity was determined as acetazolamide (AZM)‐sensitive esterase activity with 1.5 mM of p‐nitrophenyl acetate as a substrate (Gambhir et al., 2007) using a temperature‐controlled spectrophotometer (VARIAN Cary 50 Bio UV–Vis spectrophotometer). In a separate set of experiments, CA activity was measured at different temperatures in an environmentally relevant range (5–35°C) in the gill, mantle, adductor muscle, and hepatopancreas of the control mussels to characterize the tissue‐dependent capacity and temperature sensitivity (determined by apparent activation energy (*E*_a_) and Arrhenius breakpoint temperature (ABT)) of CA activity. *E*_a_ was determined from an Arrhenius plot of ln(*V*_max_) against 1/*T* (K^−1^), and ABT was determined as a point when the slope of Arrhenius plot significantly changed using an algorithm for multi‐segment linear regression (Oosterbaan, 2011).

#### 2.6.2 Calcium (Ca^2+^) and proton (H^+^) ATPases

Mantle edge tissue was homogenized and activities of Ca^2+^‐ATPase (EC 3.6.3.8) and H^+^‐ATPase (EC 3.6.3.6) were assayed as described in Ivanina et al., 2020. Inorganic phosphate (*P*_i_) was measured using malachite green assay kit (ab65622, Abcam) and ATPase activities were expressed as µmol of *P*_i_ µg protein^−1^ hr^−1^.

### 2.7 Statistical analyses

Effects of temperature, *P*_CO2_, and their interaction were assessed for all studied traits using generalized linear model (GLM) ANOVA. All factors were treated as fixed and post hoc tests (Fisher's least significant difference) were used to test differences between group means. The number of biological replicates was 5–10 for all experimental groups. Regression analysis for ABT and Arrhenius plots for CA were done using GraphPad Prism ver. 4.03 (GraphPad Software, Inc.) and SegReg software (Oosterbaan, 2011). For statistical analysis of the metabolic profiles, we used MetaboAnalyst 4.0 (Chong et al., 2019) as described elsewhere (Rebelein et al., 2018). A partial least square discriminant analysis (PLS‐DA) was used for separation of groups. Important metabolites were ranked based on Variable Importance in Projection (VIP) score of the PLS‐DA. Significantly different metabolites were identified using the Significance Analysis of Microarray (SAM) approach for high‐dimensional data analysis in small sample sizes with a Delta of 0.5 within MetaboAnalyst. SAM addresses the false discovery rate (FDR) on manifold repeated tests on the data. Significant scores are assigned by comparing the changes of each metabolite with the standard deviation of repeated measurements of a distribution estimated by random permutations (Tusher et al. 2001). A SAM plot compares the observed relative differences by the expected relative differences from the permutation results. Metabolites that showed a particular pattern were identified using PatternHunter analysis within MetaboAnalyst (Pavlidis & Noble, 2001).

Unless otherwise indicated, data are shown as means ± standard errors of means (*SEM*). Differences were considered significant if probability of type I error was < 0.05.

## 3 RESULTS

ANOVA analysis showed a significant effect of the acclimation temperature of the SMR, total lipid content of hepatopancreas, and activities of CA and Ca^2+^‐ATPase in the mantle tissues of *M. edulis* (Table 2). No significant effects of the acclimation temperature were found for glycogen levels in the hepatopancreas, ATP, ADP, AMP or AEC in the adductor muscle, or H^+^‐ATPase activity in the mantle. Acclimation *P*_CO2_ or temperature × *P*_CO2_ interactions had no significant effect on any of the studied bioenergetics‐ or biomineralization‐related traits in *M. edulis* (Table 2).

**TABLE 2:** ANOVA results of the effects of exposure temperature, *P*_CO2_, and their interaction on energy‐related indices and enzyme activities in *Mytilus edulis*

Parameter	Temperature	*P*_CO2_	Temperature × *P*_CO2_
Standard Metabolic Rate (SMR)	*F*_1,28_ = 24.97, ***p* < 0.0001**	*F*_1,28_ = 3.55, *p* = 0.07	*F*_1,28_ = 0.00, *p* = 0.98
Total Lipids	*F*_1,20_ = 16.63, ***p* = 0.0006**	*F*_1,20_ = 0.16, *p* = 0.69	*F*_1,20_ = 1.30, *p* = 0.26
Glycogen	*F*_1,35_ = 0.17, *p* = 0.68	*F*_1,35_ = 0.85, *p* = 0.36	*F*_1,35_ = 0.56, *p* = 0.45
ATP	*F*_1,20_ = 0.61, *p* = 0.44	*F*_1,20_ = 3.16, *p* = 0.09	*F*_1,20_ = 0.03, *p* = 0.86
ADP	*F*_1,20_ = 3.14, *p* = 0.09	*F*_1,20_ = 0.70, *p* = 0.41	*F*_1,20_ = 0.38, *p* = 0.54
AMP	*F*_1,20_ = 1.08, *p* = 0.31	*F*_1,20_ = 0.14, *p* = 0.71	*F*_1,20_ = 0.95, *p* = 0.34
AEC	*F*_1,20_ = 2.39, *p* = 0.13	*F*_1,20_ = 0.97, *p* = 0.33	*F*_1,20_ = 0.16, *p* = 0.69
Carbonic Anhydrase	*F*_1,20_ = 6.70**, *p* = 0.01**	*F*_1,20_ = 1.17, *p* = 0.29	*F*_1,20_ = 0.04, *p* = 0.83
Calcium ATPase	*F*_1,19_ = 9.86, ***p* = 0.005**	*F*_1,19_ = 0.01, *p* = 0.90	*F*_1,20_ = 0.18, *p* = 0.67
Proton ATPase	*F*_1,7_ = 0.03, *p* = 0.86	*F*_1,17_ = 1.13, *p* = 0.30	*F*_1,17_ = 3.25, *p* = 0.08

Note

*F*‐values are given with degrees of freedom for the factor and error in the subscript. Significant values (*p* < 0.05) are highlighted in bold.

Abbreviation: AEC, adenylate energy change.

### 3.1 Effects of warming and OA on SMR

**FIGURE 1:**
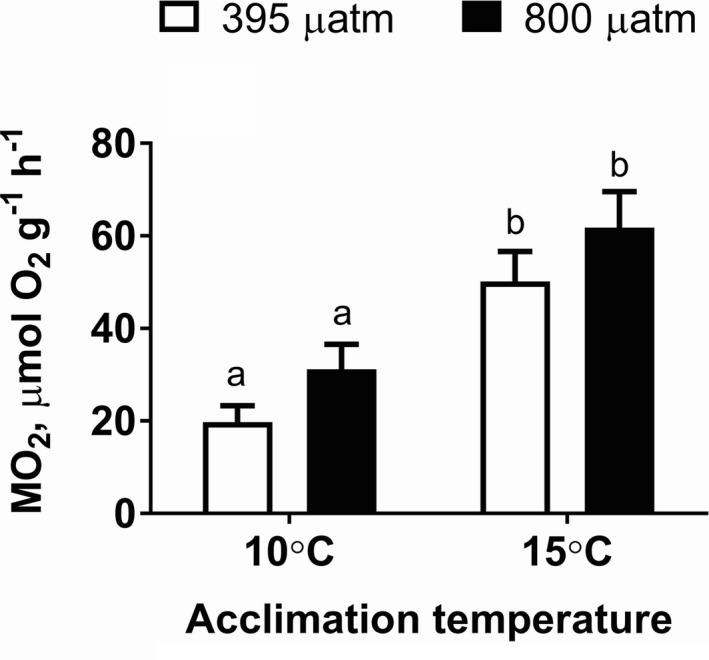
Oxygen consumption rates of *Mytilus edulis* exposed to different temperatures and *P*_CO2_. The traits were measured in mussels groups at their respective acclimation temperatures (10 vs. 15°C) and *P*_CO2_ (~400 vs. 800 µatm). Within each graph, different letters indicate means are significantly different (*p* < 0.05). Vertical bars represent *SEM*. *N* = 8–10

Warming significantly elevated SMR (*p* < 0.001) of *M. edulis* (Figure 1a; Table 2). After 4 weeks acclimation, SMR was ~2–3 times higher in mussels maintained at 15°C compared to 10°C. This effect was observed under normocapnia (*p* = 0.002) and hypercapnia (*p* = 0.009). OA did not significantly affect SMR in mussels (*p* = 0.070), although a trend of elevated SMR was observed in OA‐exposed mussels.

### 3.2 Tissue energy status under warming and OA

Warming significantly increased the total lipid content in hepatopancreas under normocapnia (*p* = 0.05) and hypercapnia (*p* = 0.001) (Figure 1b; Table 2). OA did not significantly change the lipid content in hepatopancreas, regardless of the temperature (*p* = 0.608 and 0.288 at 10 and 15°C, respectively).

**FIGURE 2:**
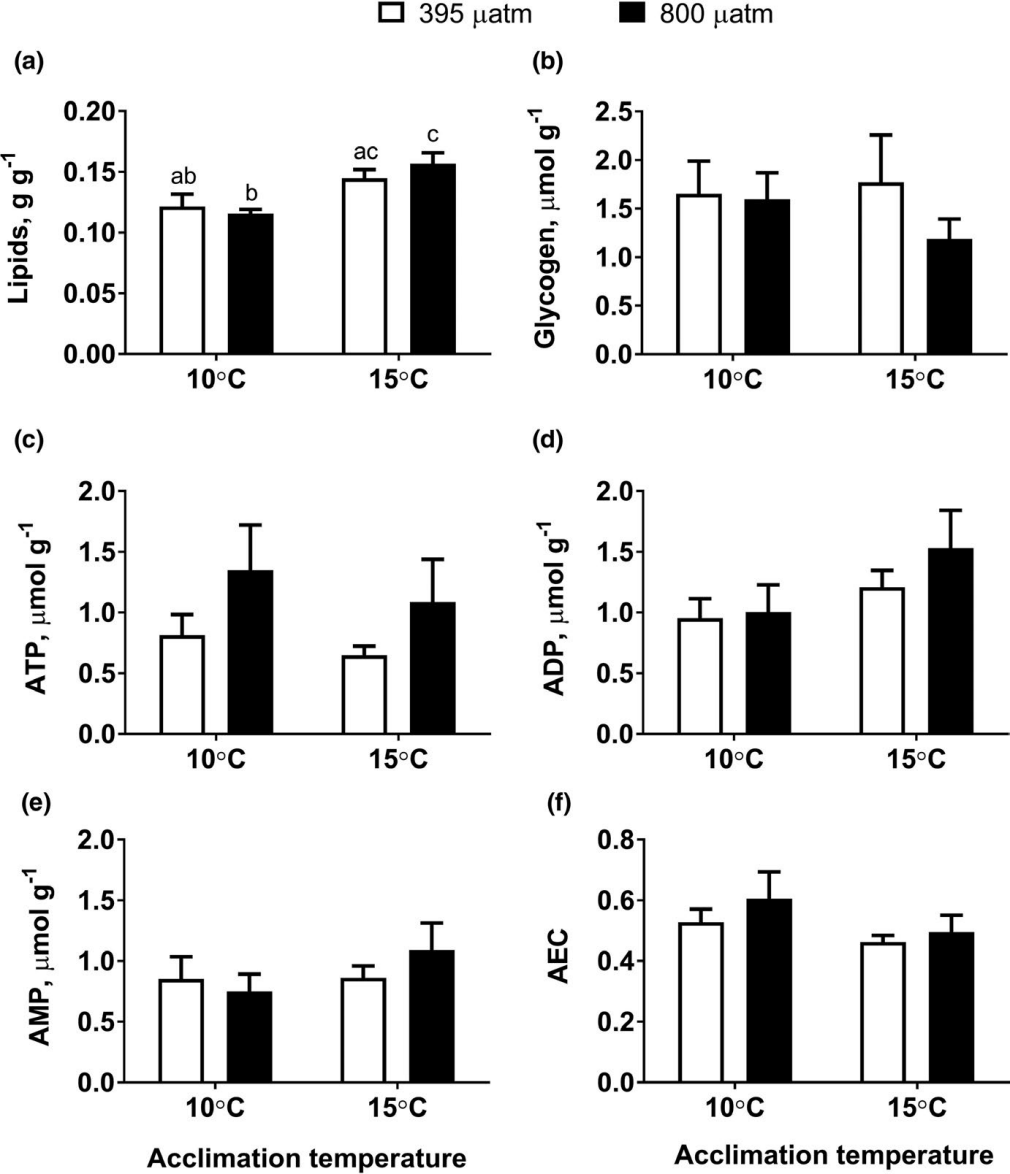
Tissue energy reserves and concentrations of adenylates of *Mytilus edulis* exposed to different temperatures and *P*_CO2_. (a) Total lipids, (b) glycogen, (c) ATP, (d) ADP, (e) AMP, and (f) adenylate energy charge (AEC). The total lipid content was measured in hepatopancreas, all other traits—in the adductor muscle. Within each graph, different letters indicate means are significantly different (*p* < 0.05). If the columns have no letters, the respective means are not significantly different between different *P*_CO2_ and temperature (*p* > 0.05). Vertical bars represent *SEM*. *N* = 6–10

No significant changes were observed for glycogen (Figure 1c) and adenylates (Figure 2) content under warming, OA, or OA combined with warming (OWA) in the muscle of *M. edulis* (Table 2).

### 3.3 Tissue‐specific shifts in metabolite profile under warming and OA

**FIGURE 3:**
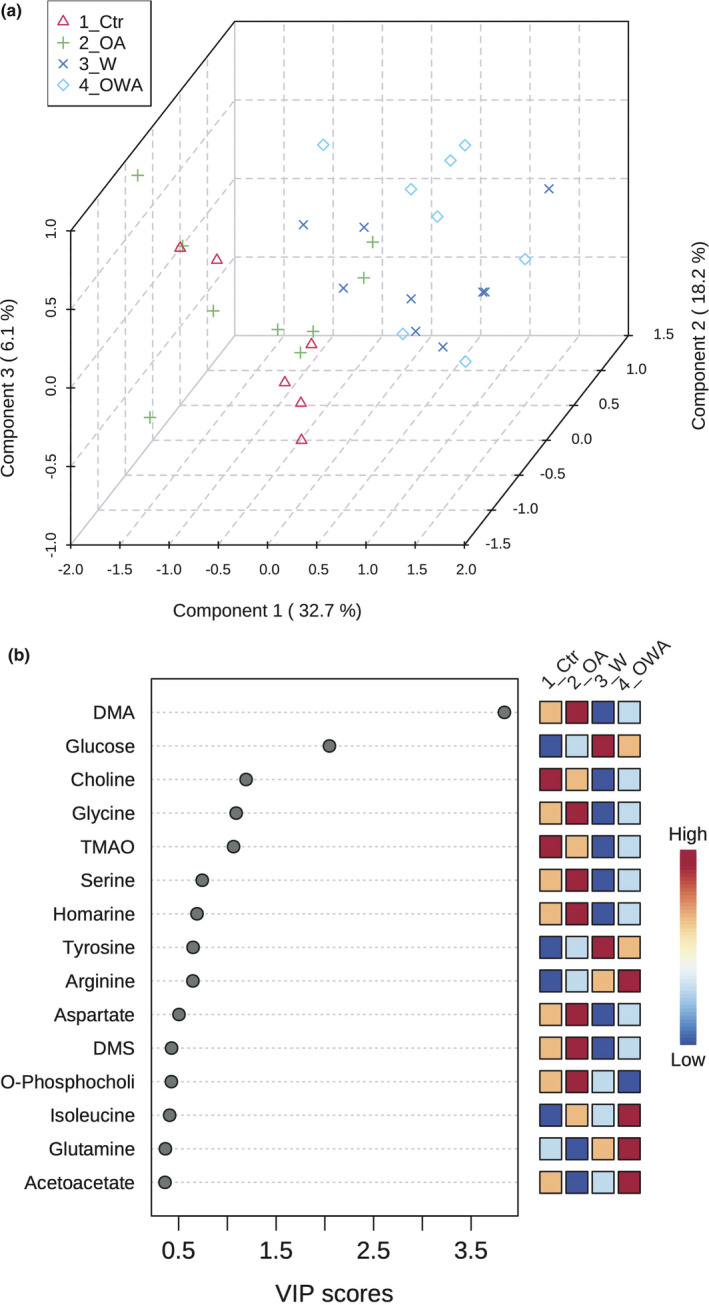
PLS‐DA analysis of metabolite profiles in the gill tissues of *Mytilus edulis* exposed to different temperatures and *P*_CO2_. (a) 3D‐loading plot of first three components separating the metabolic profiles from Warming and OWA groups (blue crosses and teal diamonds) from control (red triangles). The OA group (green plus) did not separated from control. (b) Important metabolites identified by PLS‐DA. The colored squares on the right show group‐specific relative changes in metabolite concentration. Groups: Ctr—control (acclimated at 10°C and normocapnia), OA—ocean acidification (acclimated at 10°C and hypercapnia), W—warming (acclimated at 15°C and normocapnia), OWA—ocean warming and acidification (acclimated at 15°C and hypercapnia)

**FIGURE 4:**
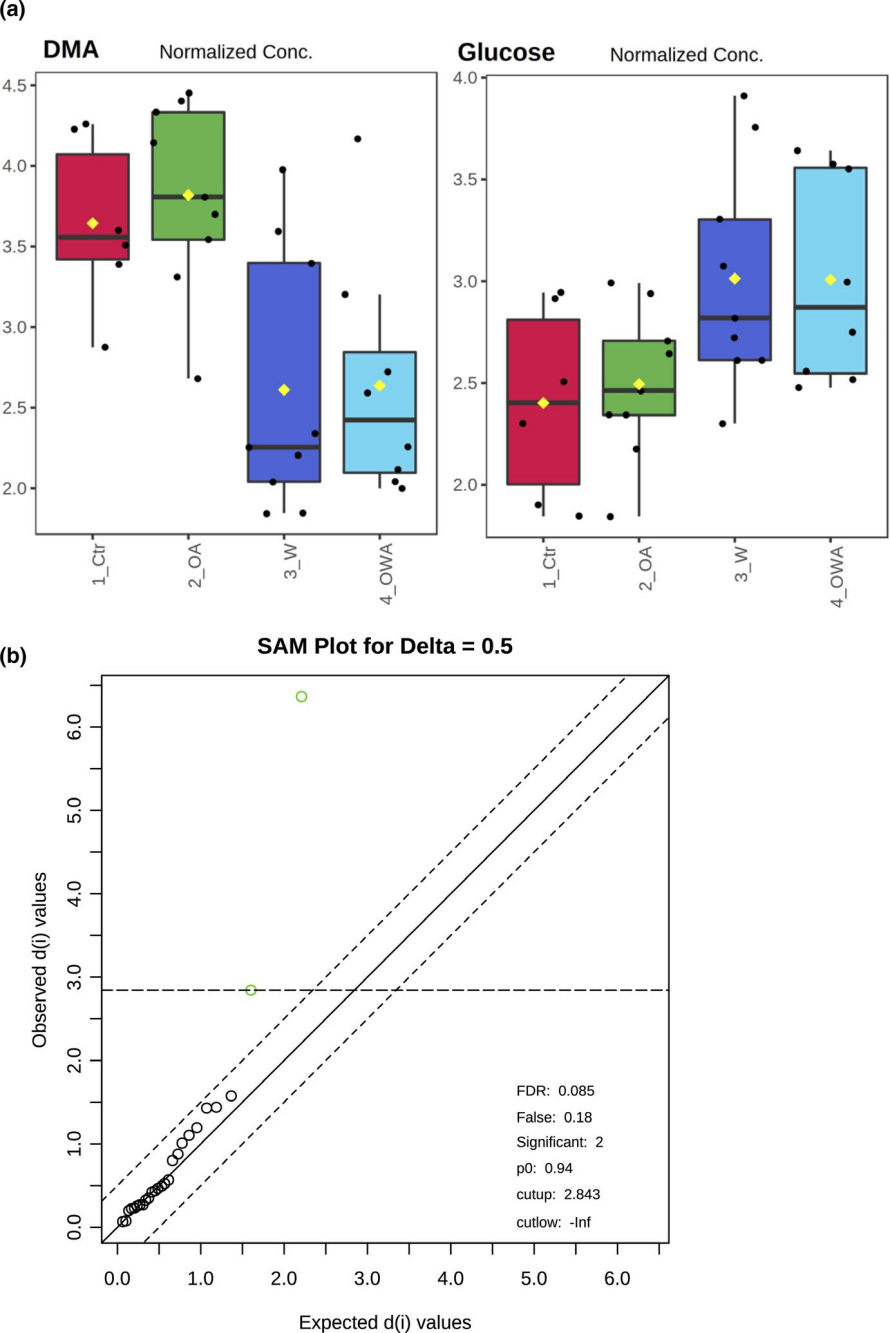
(a) Tissue levels (given in normalized concentrations) of DMA and glucose in gill tissues of *Mytilus edulis* exposed to different temperatures and *P*_CO2_. Groups: Ctr—control (acclimated at 10°C and normocapnia), OA—ocean acidification (acclimated at 10°C and hypercapnia), W—warming (acclimated at 15°C and normocapnia), OWA—ocean warming and acidification (acclimated at 15°C and hypercapnia). (b) Significance Analysis of Microarray (SAM) plot of gill tissue. Scatter plot showing observed relative differences on the axis of ordinates against the expected relative differences estimated by data permutation on the abscise using a delta of 0.5 (dotted lines). The green dots are highlighting significant differences and correspond to DMA and glucose

Untargeted NMR‐based metabolic profiling in gill and muscle of *M. edulis* revealed minor shifts in metabolite concentrations under warming, OA and OWA. In gills, PLS‐DA revealed a significant separation of 10°C‐ and 15°C‐acclimated groups (Figure 3a), indicating a temperature‐induced change in branchial metabolism. According to their VIP scores, DMA and glucose were the main metabolites leading to these differences (Figure 3b). The levels of DMA decreased and glucose levels increased under warming, regardless of *P*_CO2_ (Figure 4a). These two metabolites were also identified as significantly different between the temperature groups by SAM analysis (Figure 4b). Unlike gills, metabolite profiles of adductor muscle were not affected by temperature or *P*_CO2_ (see PLS‐DA in Figure S3).

### 3.4 Effects of warming and OA on enzyme activity

**FIGURE 5:**
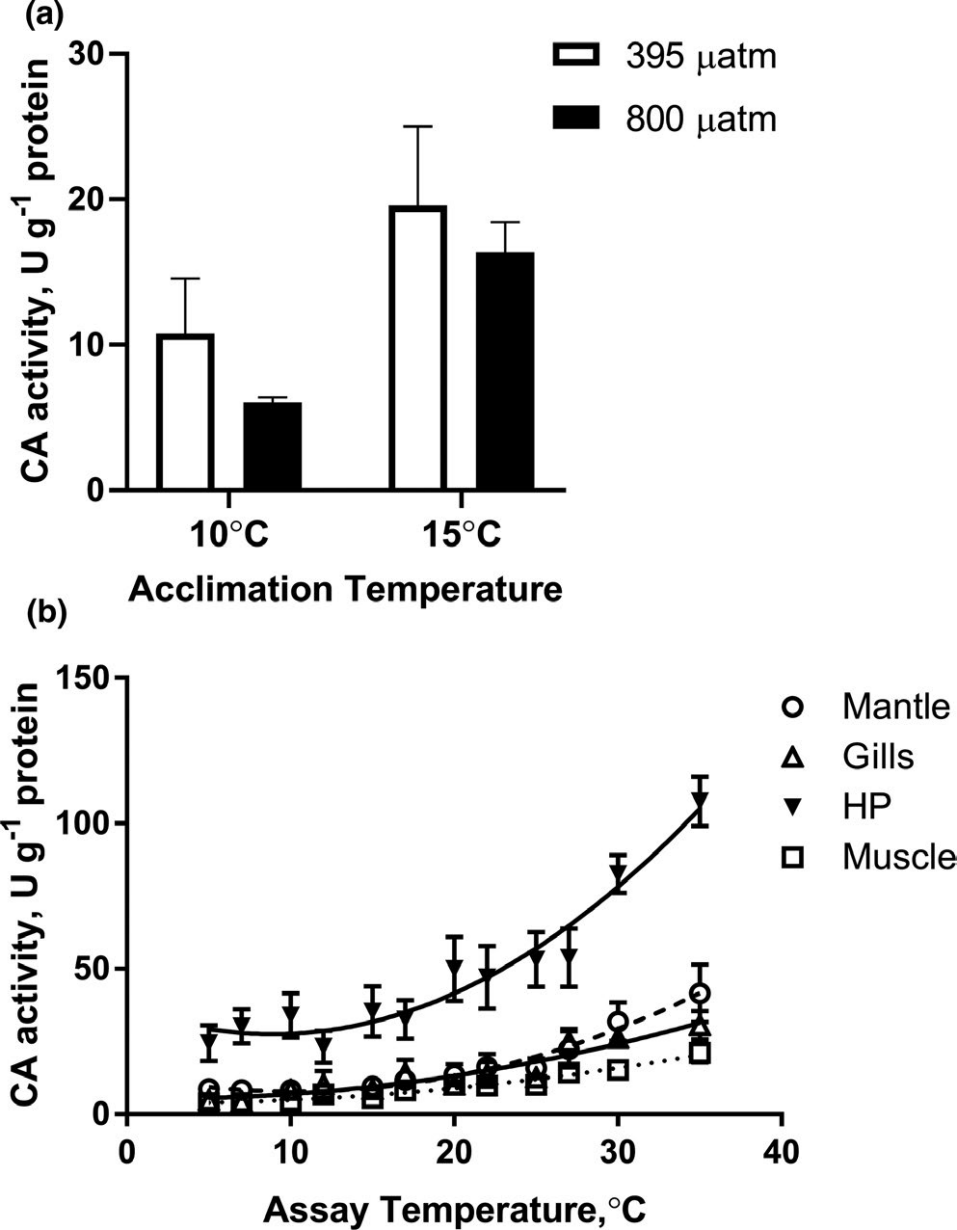
Carbonic anhydrase (CA) activity in tissues of *Mytilus edulis*. (a) CA activity in mantle edge exposed to different temperature and *P*_CO2_. (b) Tissue‐specific variation in specific activities of carbonic anhydrase with temperature. Vertical bars represent standard errors of means. HP‐hepatopancreas. If the columns have no letters, the respective means are not significantly different (*p* > 0.05). Vertical bars represent *SEM*. *N* = 5–7

Carbonic anhydrase activity in the mantle of *M. edulis* showed an elevated trend under warming (*p* = 0.001 for temperature effects) but did not under OA (*p* = 0.30) (Figure 5a; Table 2). When data for normocapnia and hypercapnia were pooled, a significant increase in CA activity at 15°C compared to 10°C (*p* = 0.018) was detected (Figure S1). Tissue‐specific CA activity over a broad temperature range (5–35°C) showed a significant effect of two‐factor (temperature x tissue) interactions (*p* < 0.0001). CA activity was significantly higher in the hepatopancreas compared to other tissues (Figure 5b). Irrespective of the tissue, CA activity monotonously increased with increasing temperatures with similar *E*_a_ (32.5–43.8 kJ/mol K^−1^) and no ABT in the studied tissues (data not shown).

**FIGURE 6:**
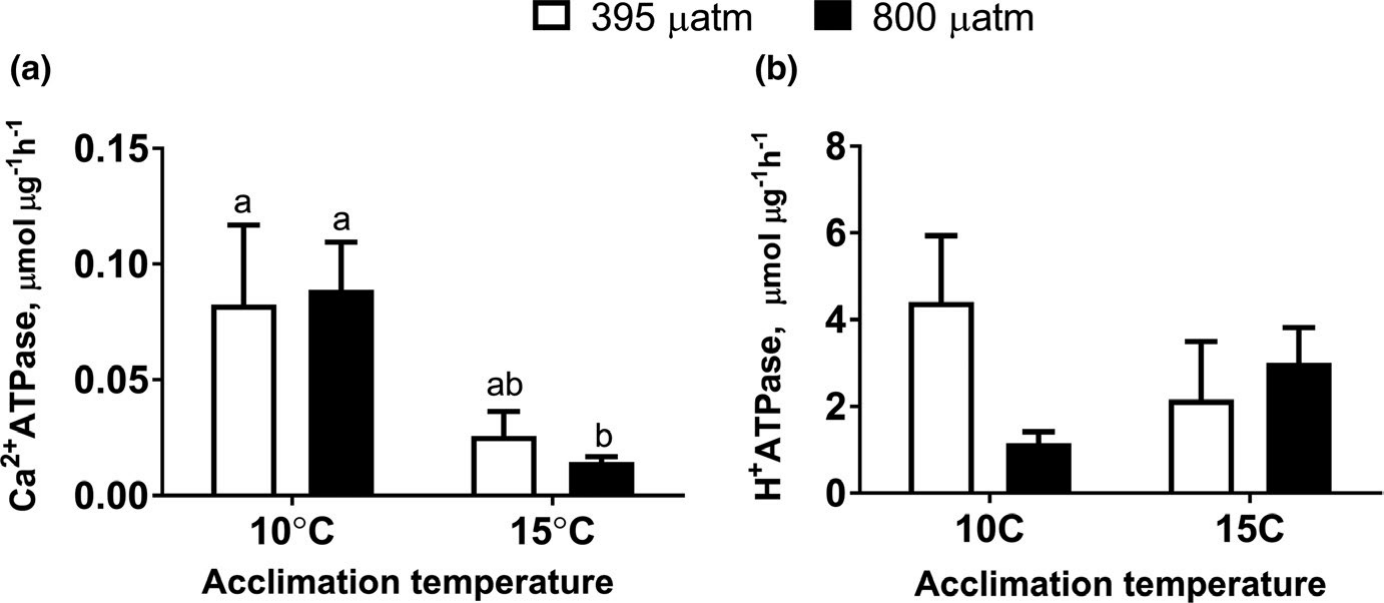
ATPases activity in mantle edge of *Mytilus edulis* exposed to different temperatures and *P*_CO2_ levels. (a) Calcium (Ca^2+^) ATPase and (b) proton (H^+^) ATPase. Within each graph, different letters indicate means are significantly different (*p* < 0.05). If the columns share a letter, the respective values are not significantly different (*p* > 0.05). Vertical bars represent *SEM*. *N* = 5–6

Ca^2+^‐ATPase activity from the mantle edge of *M. edulis* was significantly affected by warming (*p* = 0.005) but not OA (*p* = 0.905) (Figure 6a; Table 2). Warming led to a significant decrease in Ca^2+^‐ATPase activity under hypercapnia (*p* = 0.023) but not under normocapnia (*p* = 0.060).

H^+^‐ATPase activity from the mantle edge of *M. edulis* was not significantly affected by warming (*p* = 0.862) or OA (*p* = 0.303) (Figure 6b, Table 2). At 10°C, hypercapnia led to a decrease in the H^+^‐ATPase activity, but this decrease was nonsignificant (*p* = 0.06).

## 4 DISCUSSION

Our study demonstrates that adult *M. edulis* from GOM are metabolically resilient to moderate OA (*P*_CO2_ ~ 800 µatm) but responsive to warming as seen in changes in whole‐body metabolic rate, energy reserves, metabolite profiles, and enzyme activities. The combination of ocean warming and acidification (OWA) did not elicit detrimental metabolic changes in mussels beyond the effect of warming. This indicates that under these conditions, temperature is the dominant factor in determining species’ physiology. Intertidal pools, in general, are very dynamic environments with variations of temperature, pH and CO_2_ levels, where seawater pH can fluctuate from 8.1 to 6.9–6.5 (and *P*_CO2_ can change from ~ 400 μatm to more than 10,000–40,000 μatm) due to biological CO_2_ production and/or freshwater inflow, and temperature fluctuations can exceed 15–20°C during diurnal, tidal and seasonal cycles with local geographical factors moderating these variables (Burnett, 1997; Chapman et al., 2011; Ringwood & Keppler, 2002). The physiological responses of mussels in our study might be related to their ecology in GOM. There is a high temporal variability in temperature (annual SST range 15.5°C) in temperate rocky intertidal pools of GOM throughout the year (Salisbury & Jönsson, 2018). Thus, *M. edulis* in this region are predominantly exposed to large variation in temperature which might explain their plasticity to thermal stress. Earlier studies in *M. edulis* populations from thermal clines of GOM showed high phenotypic plasticity in physiology despite lack of population genetic structure and local adaptation (Lesser, 2016; Lesser et al., 2010) consistent with the findings of metabolic plasticity to warming found in our study.

### 4.1 Effect of warming and OA on bioenergetics

We observed a ~2–3‐fold increase in SMR of mussels exposed to 5°C warming under normocapnia or hypercapnia, indicating a strong temperature effect on metabolism with *Q*_10_ ~ 4–6. A 5°C increase is well within the range of temperature fluctuations experienced by mussels in GOM (Salisbury & Jönsson, 2018). Blue mussels are eurythermal and well adapted for 5–20°C range, with an upper thermal tolerance limit of ~29°C for adults (Gosling, 1992). Therefore, temperatures in our study are within the ecological relevant and even optimal range for this species. On the other hand, Pejus temperatures represent thermal transitions where organisms mount compensatory mechanisms that divert away from performance parameters with direct fitness correlates (Zittier et al., 2015). Previous studies have shown that the circulatory system of *M. edulis* reached its temperature‐induced capacity limit above 25°C, indicating the onset of the Pejus range at this temperature (Zittier et al., 2015). The elevated SMR at 15°C seen in the present study, therefore, reflects the rate‐enhancing effects of temperature on physiological and biochemical reactions such as activities of metabolic enzymes, ion channels, mitochondria, and other important bioenergetic processes (Hochachka, 1973). Recent meta‐analysis also indicates that temperature threshold for long‐term survival of *M. edulis* is ~20°C (Lupo et al., 2021). Therefore, 20°C appears to be close to the metabolic optimum of *M. edulis*, so rate‐enhancing effects of temperature dominate over the potentially negative impacts on metabolism as long as warming occurs below the 20°C threshold, as in our study.

Unlike temperature effect, modest OA had no effect on SMR regardless of the temperature. Metabolic response to OA in *M. edulis* can vary and depend on magnitude of *P*_CO2_, food availability, and population (Fitzer et al., 2014; Hüning et al., 2013; Lesser, 2016; Thomsen et al., 2013; Thomsen & Melzner, 2010; Zittier et al., 2015). Fitzer et al. (2014) showed that for *M. edulis* at 1,000 µatm and beyond, biomineralization continued but with compensated protein metabolism and shell growth indicating that ~1,000 µatm could be an OA metabolic tipping point for *M. edulis*. In our study, *P*_CO2_ levels were below this threshold and could explain the physiological tolerance of mussels seen here. Our hypothesis that *P*_CO2_ could exacerbate the increase of SMR caused by elevated temperature was not supported in this study. The outcome of OWA on metabolic rate in bivalves is commonly additive; albeit in other cases, the effects of temperature or *P*_CO2_ dominate (see Lefevre, 2016 and references therein). Furthermore, metabolic responses of bivalves to OWA are dependent on the degree of temperature or *P*_CO2_ stress. For example, Lesser, 2016 showed that mussels from GOM showed metabolic depression as a protective response when exposed to combined stress of higher warming (22°C) and modestly elevated *P*_CO2_ (560 µatm).

Temperature (but not OA) had a marked effect on lipid content in *M. edulis*. We observed an increase in lipids under warming in hepatopancreas, under both normocapnia and hypercapnia. In bivalves, lipids are primarily stored in hepatopancreas (Giese, 1966) and synthesis, storage, and use of lipids show pronounced seasonal cycles. Specifically, lipids are accumulated during summer (at high temperature and food availability) and used for metabolism and initiation of gametogenesis during winter (at low temperature and food availability) (Fokina et al., 2015). Laboratory studies indicate that lipid accumulation is a direct response to temperature in mussels (Fokina et al., 2015; Wu et al., 2021) and might reflect a metabolic adjustment for anticipated reproduction (which requires high energy investment as well as lipid deposition into developing gametes) in mussels.

Unlike lipids, the glycogen content did not change in response to warming in mussels from GOM. *M. edulis* from Baltic Sea also showed no change in glycogen content during warming from 10 to 15°C and from 15 to 20°C (Wu et al., 2021). Modest hypercapnia likewise had no effect on the glycogen content of adductor muscle of mussels in our present study. Earlier studies show that impacts of temperature and OA on glycogen reserves of mussels are threshold dependent. Thus, mussels from GOM showed a marked decrease in the glycogen content of adductor muscle when exposed to higher temperature (22°C) alone and combined with modest acidification (560 µatm *P*_CO2_, pH 7.9) (Lesser, 2016). The concentrations of adenylates and AEC in adductor muscle remained at steady‐state levels in all exposures, indicating that cellular energy balance in mussels was maintained under all temperatures and OA scenarios. Overall, our findings indicate that temperature and OA used in our present study are not energetically stressful to GOM mussels (at least under *ad libitum* feeding conditions).

Warming alone or OWA altered the metabolite profile of *M. edulis* in a tissue‐dependent manner. Warming from 10 to 15°C increased glucose and decreased dimethyl amine (DMA) in the gills, whereas adductor muscle metabolites were not affected. Increased glucose could reflect mobilization of energy reserves to meet increased tissue energy demand. Furthermore, warming could increase glucose levels via gluconeogenesis by channeling of amino acids like serine, alanine, and glycine into pyruvate and increased activity of the enzyme phosphoenolpyruvate carboxykinase (PEPCK) (Ellis et al., 2014; Le Moullac et al., 2007). In our study, we saw a trend for decreased glycine and serine in gills under warming and OWA, suggesting a potential for increased flux through gluconeogenesis that warrants further investigation. The decrease in DMA, a common organic osmolyte found in gills of bivalves (Zhang et al., 2011), might osmotically compensate for elevated glucose in *M. edulis* gills during warming.

In *M. edulis*, OA had no effect on the metabolite profile in gills or muscle tissue. Similarly, metabolite profiling studies with *P*_CO2_ ≤ ~1,000 µatm (pH ≥ ~7.8) reported no OA‐induced alteration in metabolite levels of bivalves (Dickinson et al., 2012; Ellis et al., 2014; Wei et al., 2015) whereas higher *P*_CO2_ (≥1,500 µatm, pH ≤ ~7.7) led to a shift in metabolite profiles with alterations in energy metabolism (Ellis et al., 2014; Lannig et al., 2010; Wei et al., 2015). These findings are consistent with the notion that modest OA is not a metabolic stressor for *M. edulis*, and the studied GOM population follows this general pattern.

### 4.2 Effect of warming and OA on enzyme activity

In mollusks, CA plays a key role in the maintenance of acid–base homeostasis of all tissues as well as biomineralization in the mantle (Li et al., 2016; Ramesh et al., 2020; Wang et al., 2017). In *M. edulis*, CA activity increased with acute warming (5–35°C) in all tissues with similar *E*_a_ values indicating a concerted whole‐body response of this enzyme to warming. No Arrhenius breakpoint temperature (ABT) was found for CA activity from 5 to 35°C indicating high thermal tolerance of this enzyme. We found highest CA levels in hepatopancreas as compared to other tissues (gills, muscle and mantle) in *M. edulis*. This might reflect differences in overall metabolic activity (and therefore, different metabolic CO_2_ and proton loads) among the tissues that require different levels of CA to maintain acid–base balance. Acclimation to 15°C upregulated CA activity in a key biomineralizing tissue (the mantle edge) of *M. edulis*. Similarly, long‐term (15 weeks) exposure to elevated temperature (27°C) led to a notable increase in CA activity in bivalves *Crassostrea virginica* and *Mercenaria mercenaria* (Ivanina et al., 2013). This increase is likely linked with the overall increase in metabolic rates at elevated temperatures and can assist with shell deposition.

CA activity remained unchanged in response to OA in mantle edge of *M. edulis*. Previous studies showed variable CA responses to OA in bivalves including mussels (Ivanina et al., 2020). Similar to our findings, CA activity in the mantle of *C. virginica* and *M. mercenaria* remained unchanged after exposure to elevated *P*_CO2_ (800 µatm) for 2–15 weeks (Ivanina et al., 2013). In contrast, CA activity in mantle of *M. edulis* decreased after prolonged (6‐months) exposure to elevated *P*_CO2_ (750 µatm) exposure (Fitzer et al., 2014). In oysters, CA accumulated along mantle edge in response to *P*_CO2_ exposure (2,622 µatm, pH 7.50), suggesting an active role of CA in ion‐regulation and acid–base balance (Wang et al., 2017). However, the effect of OA on CA activity is threshold dependent. At *P*_CO2_ < 1,000 µatm mussels try to compensate for intracellular acid loads instead of decreasing their metabolism (Fitzer et al., 2014; Hüning et al., 2013; Thomsen & Melzner, 2010). Taken together, these data suggest that *M. edulis* in the GOM can upregulate acid–base balance contributing to their metabolic plasticity toward warming, but moderate OA has no effect on this trait.

As a consequence of maintaining acid‐base balance, OA may change concentrations of H^+^, HCO_3_^−^, Ca^2+^, Mg^2+^, and Cl^−^ in calcifiers (Fitzer et al., 2014; Ramesh et al., 2017). Ion transport is an important contributor to energy budget of biomineralization because Ca^2+^ transport and removal of excess protons from the site of biomineralization are ATP‐dependent (Ivanina et al., 2020). In GOM mussels, activity of Ca^+^‐ATPase and H^2+^‐ATPase‐in the mantle remained stable under moderate warming and OA (except for a modest but significant decline in Ca^2+^‐ATPase under OWA). This indicates that the mussels can maintain ion regulatory fluxes at least in the mantle edge despite variations in temperature and *P*_CO2_ relevant to near‐future climate change. Mussels from other environments acidified like Kiel Fjord (pH < 7.5) also build and maintain their shells despite fluctuating *P*_CO2_ and pH, partially owing to enhanced ion transport (Thomsen et al., 2013, 2017). Furthermore, when mussel larvae were raised under OA between 500 and 1,500 µatm *P*_CO2_, ∆H^+^ at the calcification site when compared to seawater remained constant, irrespective of *P*_CO2_ (Thomsen et al., 2013). In *C*. *virginica* and *M. mercenaria* activities of Ca^2+^ ATPase and H^+^ ATPase, as well as the cellular energy costs of Ca^2+^ and H^+^ transport in the biomineralizing cells (mantle and hemocytes) were insensitive to ocean acidification (pH 7.8) (Ivanina et al., 2020). This indicates that intertidal species (such as mussels, oysters, and clams) that are adapted to variable temperature and pH in their habitat are generally tolerant against moderate warming and OA, predicted by the climate change models.

## 5 CONCLUSIONS

In this study, we report that adult *M. edulis* from GOM are sensitive to warming but tolerant to moderate acidification scenario predicted by IPCC for the year 2100. This result also provides an insight in the natural history of GOM mussels given that in the last decade (2005–2014) GOM was characterized by an extreme warming trend (Salisbury & Jönsson, 2018). Although this study is limited to adults and does not consider larval stage sensitivity of *M. edulis*, our results support earlier reports that acidification scenarios for the next 100–300 years do not affect this species (Telesca et al., 2019). Taken together, our study provides important data about extant levels of plasticity in physiology of mussels as well as insights into potential sensitivity of mussels to future global change.

## CONFLICT OF INTEREST

The authors declare no conflict of interest.

## AUTHOR CONTRIBUTIONS

**Omera B. Matoo:** Conceptualization (lead); formal analysis (lead); investigation (lead); methodology (lead); writing – original draft (lead); writing – review and editing (lead). **Gisela Lannig:** Data curation (equal); formal analysis (equal); visualization (equal); writing – review and editing (supporting). **Christian Bock:** Data curation (equal); formal analysis (equal); visualization (supporting); writing – review and editing (supporting). **Inna M. Sokolova:** Conceptualization (equal); funding acquisition (lead); methodology (equal); project administration (lead); resources (lead); writing – review and editing (supporting).

## Supporting information

Figure S1‐S3

## Data Availability

Supplemental files are available at FigShare. Phenotypic data is deposited in the Dryad Digital Repository (https://doi.org/10.5061/dryad.ffbg79ctf).
